# Individual and generalized lower limb muscle activity and kinematic adaptations during running on an unpredictable irregular surface

**DOI:** 10.1186/1757-1146-7-S1-A2

**Published:** 2014-04-08

**Authors:** Charlotte Apps, Rui Ding, Jason Tak-Man Cheung, Thorsten Sterzing

**Affiliations:** 1 Sports Science Research Center, Li Ning (China) Sports Goods Co Ltd, Beijing 101111, China; 2 School of Sport and Exercise Sciences, Liverpool John Moores University, Liverpool, L3 3AF, UK

## Background

Natural surfaces are irregular but only limited studies have researched their effect on gait because of the predominantly flat surfaces where measurements are taken [1]. Regularly, biomechanical research also tends to group mean results of many participants together to find the generalised response to a constraint. This often masks individual adaptation strategies [2]. Therefore, the purpose of this study was to analyse biomechanical responses during running on an unpredictable irregular surface (UIS), at the individual level.

## Methods

Seventeen healthy, male participants ran on a treadmill at 8 km/h with a predictable regular surface (PRS) and an UIS, created by attaching EVA dome shaped inserts of two different heights and hardnesses. The mean and standard deviation, as a measure of variability, were calculated for lower limb kinematics and electromyography of five selected muscles of the left leg for 16 steps. Single parameters between individuals were compared, and additionally group results between the treadmill surfaces were obtained by Wilcoxon signed ranked tests (p<.05).

## Results

**Figure 1:**
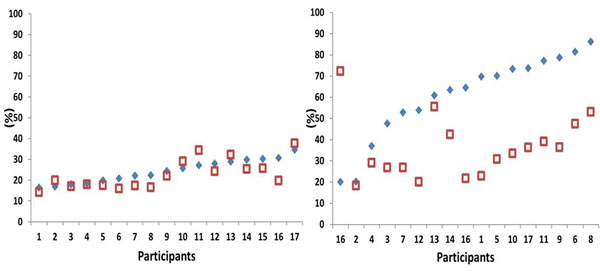
Normalized mean EMA of tibialis anterior: individual response during stance phase (left) indicated by no systematic relationship between surfaces, generalized group response during latency period (right) indicated by increased EMA on UIS for all participants. Squares denote irregular surface, diamonds regular surface.

There were individual responses to UIS in mean EMG muscle activation (EMA) for four out of the five leg muscles (vastus medialis, biceps femoris, tibialis anterior and gastrocnemius medialis) and variability of EMA (bicep femoris, tibialis anterior, gastrocnemius and peroneus longus) during total stance phase. However, within the latency period (first 30 ms after touchdown) the mean EMA was no longer individual in the vastus medialis and tibialis anterior; there was a common group increase and decrease respectfully on UIS (Figure 1). Contrastingly, the response of the peroneus longus during the latency phase was individual, with participants applying either increased or decreased EMA strategies for frontal plane ankle control. Sagittal plane shoe, ankle, knee and hip touchdown kinematic characteristics were directly affected by the surface constraint as they were common between participants throughout. Variability of the kinematics on UIS was not individual and increased regardless of joint and stance phase period analysed.

## Conclusion

Individual lower limb muscle activation strategies, accompanied by common group sagittal plane joint angles at touchdown occurred during running stance phase on UIS. Due to the nature of the UIS, it remains unknown whether personal muscular control was triggered by the different perturbation experienced or executed proactively by runners. For better understanding of adaptations to shoe-surface interactions, next to searching for common neuro-muscular patterns, more focus should be placed on analyses of individual responses and the sub-periods of stance phase.
